# Upper body compression wear improves muscle oxygenation following intense video game training: a randomized cross-over study among competitive gamers

**DOI:** 10.1186/s13102-023-00720-5

**Published:** 2023-09-11

**Authors:** Joanne DiFrancisco-Donoghue, Alexander Rothstein, Min-Kyung Jung, Hallie Zwibel, William G Werner

**Affiliations:** 1 Department of Osteopathic Medicine, New York Institute of Technology/Academic Health Care Center, College of Osteopathic Medicine (NYITCOM), Northern Blvd, PO Box 8000, Old Westbury, NY 11568 USA; 2 Department of Interdisciplinary Health Sciences, New York Institute of Technology (NYIT), Old Westbury, NY USA; 3 Department of Research, NYITCOM, Old Westbury, NY USA; 4 Department of Family Medicine, NYITCOM, Old Westbury, NY USA; 5 School of Health Professions, Department of Physical Therapy, New York Institute of Technology (NYIT), Old Westbury, NY USA

**Keywords:** Muscle oxygen, Fatigue, Graduated compression, Esports

## Abstract

**Background:**

Esport players require a high number of action moves per minute to play, with substantial contractions of the wrist extensor muscles. Players frequently suffer from acute fatigue. The purpose of this study was to examine the use of below the elbow compression sleeves on Sm02 during intense aim training. Secondly, to examine players’ performance and perception with and without compression.

**Methods:**

This study was conducted at the New York Institute of Technology and enrolled fifteen collegiate esport players, 2 women and 13 men (age 21.2 ± 2.2). All subjects signed written consent. Participants performed 3 high intensity bouts of an aim trainer followed by a 15-minute rest before doing another 3 bouts of high intensity training conducting the other arm of the study. The compression wear order was randomized. The primary outcome included Sm02 of the extensor carpi radialis longus using near-infrared spectrometry. Secondary outcomes included Kills Per Second (KPS), Score, Total Time to Kill (TTK), accuracy, and perceived performance.

**Results:**

Following 15 min of recovery, there was a significant rise in Sm02 while wearing the compression sleeve compared to no compression sleeve (p = 0.004). No change in Sm02 was seen while gaming. In trials 1 and 2, wearing the compression sleeve resulted in a significant increase in KPS and score when compared to not wearing it (p = 0.002,0.006). Although TTK and accuracy did not alter, 46.7% of participants believed the compression sleeve aided their performance.

**Conclusions:**

This study provides support that wearing below the elbow upper body compression sleeves while performing high intensity gaming may reduce fatigue, improve muscle recovery and gaming performance.

**Trial registration:**

Clinicaltrials.gov identifier NCT05037071. Registered 08/09/2021. URL: Arm Compression on Muscle Oxygen Saturation - Full Text View - ClinicalTrials.gov

## Background

Muscle deoxygenation and reoxygenation has been studied in multiple athletic populations [1–3]. Muscle oxygen saturation (SmO2) is an indirect measure of muscle efficiency, which is defined as the balance between the rate of oxygen consumption by the muscle and the rate of oxygen (O2) being replenished in the muscle.1) It is important to all athletic populations, including both endurance and power athletes, as it is a marker for how efficient a muscle is during performance as well as recovery [1–3]. If the supply does not meet the demand of the muscle, then the muscle metabolism becomes increasingly anaerobic which can lead to rapid fatigue [1, 4].

A competitive esport player can perform up to 600 mouse and keyboard actions per minute (APM) on a typical training day [5]. A routine training day for a competitive esport player can range from 5 to 10 h of play with no break [5]. In comparison, office workers perform an average of 130–180 keyboard and mouse inputs per minute over the course of an 8-hour work day [6]. These APMs require sustained wrist extension in conjunction with repetitive forearm muscle contractions in multiple planes, as well as shoulder stability and postural stability. With these fine motor demands, it is common for players to suffer from acute fatigue and chronic overuse potentially resulting in wrist and arm injuries [7].

The use of compression wear has expanded from clinical use into the sports market. Athletes in various sports wear compression garments with the assumption that it will improve performance and facilitate muscle recovery. The recommendations to wear compression gear in athletes is based on an assumption of improvement in venous blood flow which improves exchange of fresh blood and blood waste [1, 3]. Anecdotally, Lebron James and Ray Allen are among many National Basketball Association (NBA) players that wear upper compression regularly when competing, as well as professional Major League Baseball (MLB) player Shohei Ohtani. In the 2016 Olympics, it was estimated that 90% of athletes used some form of compression performance gear [8].

Compression wear during endurance exercises has mostly been studied using lower body compression, with conflicting results [1, 9–11].There is minimal evidence supporting the use of compression sleeves during intermittent high-intensity exercise, and even less so for the use of upper body compression sleeves [9, 12–15]. However, it has been demonstrated that forearm compression sleeves enhance arterial blood flow [16]. Therefore, the performance and recovery of esport players may be improved by this increased blood flow; hence this enhanced blood flow may be beneficial to their performance.

Most modern compression gear marketed toward athletes use garments that employ ‘graduated compression’. This means that the highest amount of pressure is on the most distal parts of your body (e.g. ankles if you are using lower body compression, wrists if using upper body compression) and the pressure gradually reduces as it moves up toward your body. Compression wear varies in pressure range. The amount of compression is measured in mmHg and light compression can range from 18 to 21 mmHg, moderate 23–32 mmHg, strong 34–46 mmHg and > 49 mmHg very strong [17]. Most over-the-counter athletic compression garments range from 18 to 21 mmHg.

Considering esports research is in its early stages, oxidative capacity of the finger and wrist extensors during prolonged video gaming has never been explored. The aim of this study is to evaluate the use of upper body graduated compression gear on Sm02 during high intensity first person shooter (FPS) game training. Secondly, a goal is to examine players’ performance with and without upper body graduated compression, and lastly to examine perception and comfort level while playing with or without compression.

## Methods and procedures

**Fig. 1:**
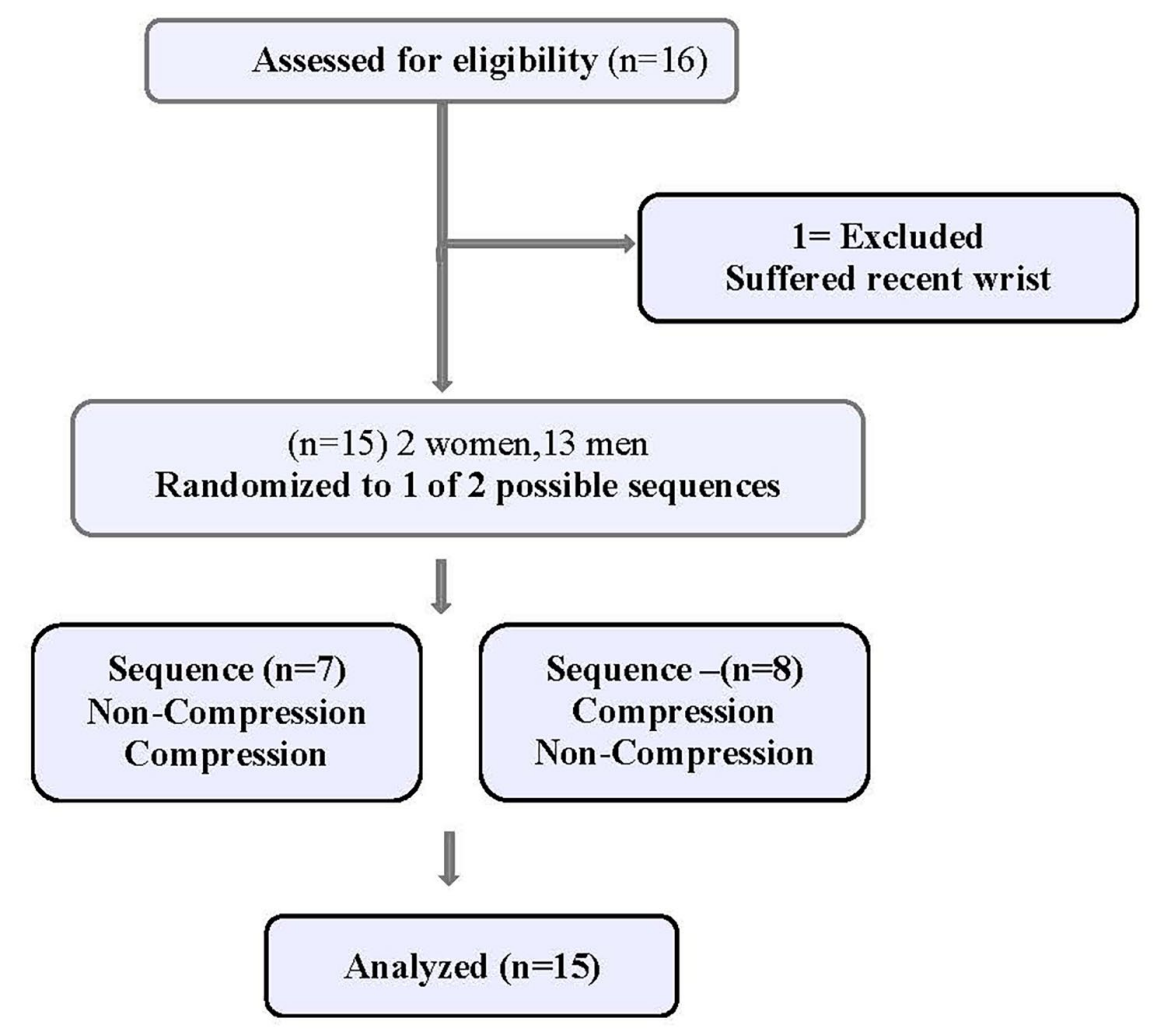
Study Flow Diagram

This study was approved by the New York Institute of Technology (NYIT) Institutional Review Board and registered on Clinicaltrials.gov identifier NCT05037071. Fifteen healthy collegiate esport players, 2 women and 13 men (mean age 21.2 ± 2.2), signed written informed consent prior to participation in this study (Fig. 1). Inclusion criteria included: (1) A ranked esport player with over 500 h in their game (self-reported); (2) Non-smoker; (3) No history of heart disease, pulmonary disease, diabetes, or other metabolic disease; Exclusion criteria included: (1) Use of any prescribed or over the counter medications that would influence metabolic outcomes or blood viscosity; (2) Any prior injury to the dominant upper arm within the past year. (Table 1)

**Table 1:** Demographics

	n = 15
Age (SD)	21.2 (2.2)
Weight.Kg (SD)	70.5 (12.2)
Height.inches (SD)	71(4.5)
BMI (SD)	20.9(4.3)
Men (%)	86.7
Right-handed (%)	86.7
Ethnicity (%)	
African American	20
Asian	33.3
Caucasian	40
Other	6.7
Primary Game (%)	
Halo	6.7
Arma 3	6.7
Valorant	33.3
Overwatch	20
Rocket League	6.7
Dragon Ball Fighterz	6.7
League of Legends	6.7
Casual Hours Played Weekly (%)	
under 1 h	13.3
1–2 h	6.7
3–4 h	26.7
5–6 h	13.3
more than 6 h	40
Competetive Hours Played Weekly (%)	
1–2 h	40
3–4 h	33.3
5–6 h	26.7

### Procedures

This study was a randomized cross-over design which entailed subjects coming to the NYIT esport gaming lab in Old Westbury, New York for 1 testing day. Subjects arrived at least one hour post prandial. The room was kept temperature controlled within 2–3 degrees of 21 degrees Celsius each testing day.

Testing was randomized by subject in order of sequence regarding use of compression garment(intervention) versus no compression (control) garments using an online sequence generator. (RANDOM.ORG - Sequence Generator)

### Sm02

The extensor carpi radialis longus muscle in the forearm is one of the primary wrist extensor muscles involved in console gaming by facilitating wrist extension during camera control, aiming, button pressing, and precise movements. During gaming sessions, players often need to perform these repetitive and precise movements with their thumbs and fingers while also manipulating the controller with their wrists. The extensor carpi radialis longus is easily palpable at one third of the line between the lateral epicondyle and the styloid process of the radius [13, 14]. This was the location the Sm02 sensor was placed. Near infrared spectroscopy (Moxy™, Minnesota, USA) was used to assess Sm02. Near infrared spectroscopy measures the ratio of the oxyhemoglobin concentration to the total hemoglobin concentration in the muscle in real time and reports it as a percentage, which is indicated as muscle oxygen saturation or muscle oxygenation (SmO2) [18, 19].

### FPS training

This study used a Gridshot aim trainer to implement FPS intense training. A large problem in current esport literature is the lack of validated outcome measures due to the variability of each game and the level of competition someone may compete against. The only way to consistently keep the level uniform across all conditions and to quantify how many action moves the player performed with their finger was to use a standardized aim trainer. Using Gridshot, the following outcome measures were collected for performance: Kills Per Second (KPS), Time to Kill (TTK), and Accuracy.

The Moxy sensors were taped into place. Pre-gaming measurements were conducted following 10 min of complete rest. Each subject then played an 8-minute bout of State Space Lab (STATE SPACE LABS, INC. New York NY, Statespace) Gridshot AIM trainer with 1 min rest between training sets. This was repeated 2 more times for a total of 24 min of training.

Dependent on random assignment, participants either played with or without compression. Under the compression condition a Juzo™ pressure monitor (Compression Innovations Inc. Cuyahoga Galls, OH, USA) was inserted under the compression garment to measure distal and proximal mmHg of pressure and the pounds per square inch were recorded.

The subjects then rested for 15 min before switching to the other condition of the study where they repeated the same three bouts of Gridshot for 8 min with 1 min rest between each bout. During this time muscle Sm02 and heart rate were monitored continuously. Re-oxygenation during recovery was calculated at 5 min, and 15 min post training. Heart rate was collected using Wahoo™ Optical Heart Rate armbands with Bluetooth (Wahoo Fitness™, Atlanta, GA, USA). The Moxy units and the Wahoo HR monitors both were synced using PerfPro Studio® software (Vision Quest Virtual, LLC, Illinois). (Table 2)

**Table 2:** Protocol

Protocol	Minutes
Rest	10:00
AIM trainer	8:00
Rest	1:00
AIM trainer	8:00
Rest	1:00
AIM trainer	8:00
Recovery	5:00
Recovery	15:00

Repeat with other condition

### Compression

#### Measuring for proper upper body compression

**Fig. 2:**
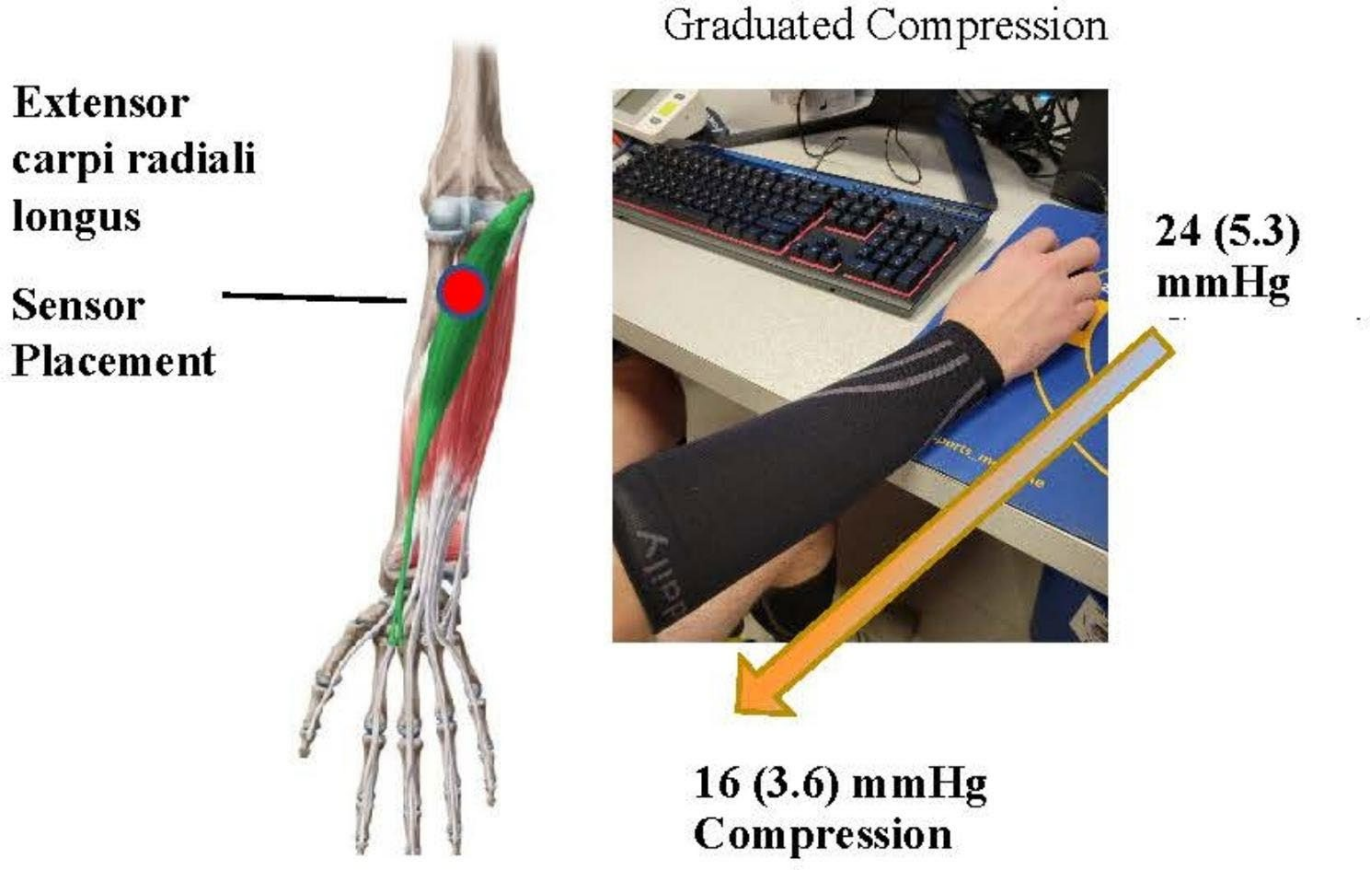
Sensor placement and graduated compression sleeve

This study used graduated compression garments that fit below the elbow. The dominant arm (arm manipulating the mouse) was fitted according to manufacturer instructions. (Fig. 2)

### Statistical analysis

IBM SPSS V.27 was used to carry out all statistical analyses. Statistical significance for this study was set at p ≤ 0.05. A two-way repeated measures analysis of variance was conducted to compare SmO2 and HR at rest, the final values of the 3rd trial (minute 24 of Gridshot training), 5-minute recovery, and 15-minute recovery following each arm and for all performance outcomes. A post hoc analysis was conducted when significance was found. Significance was set at p ≤ 0.05.

## Results

### Gaming and recovery

There was no change in Sm02 while actively gaming or at rest between trials 1, 2 and 3 while wearing the arm compression sleeve compared to not wearing the compression (p value = 0.18,0.11, 0.47,0.72). There was no change in HR in either condition (p = 0.35). There was no significant difference in HR at rest or throughout gaming (p = 0.59).

### Recovery

**Fig. 3:**
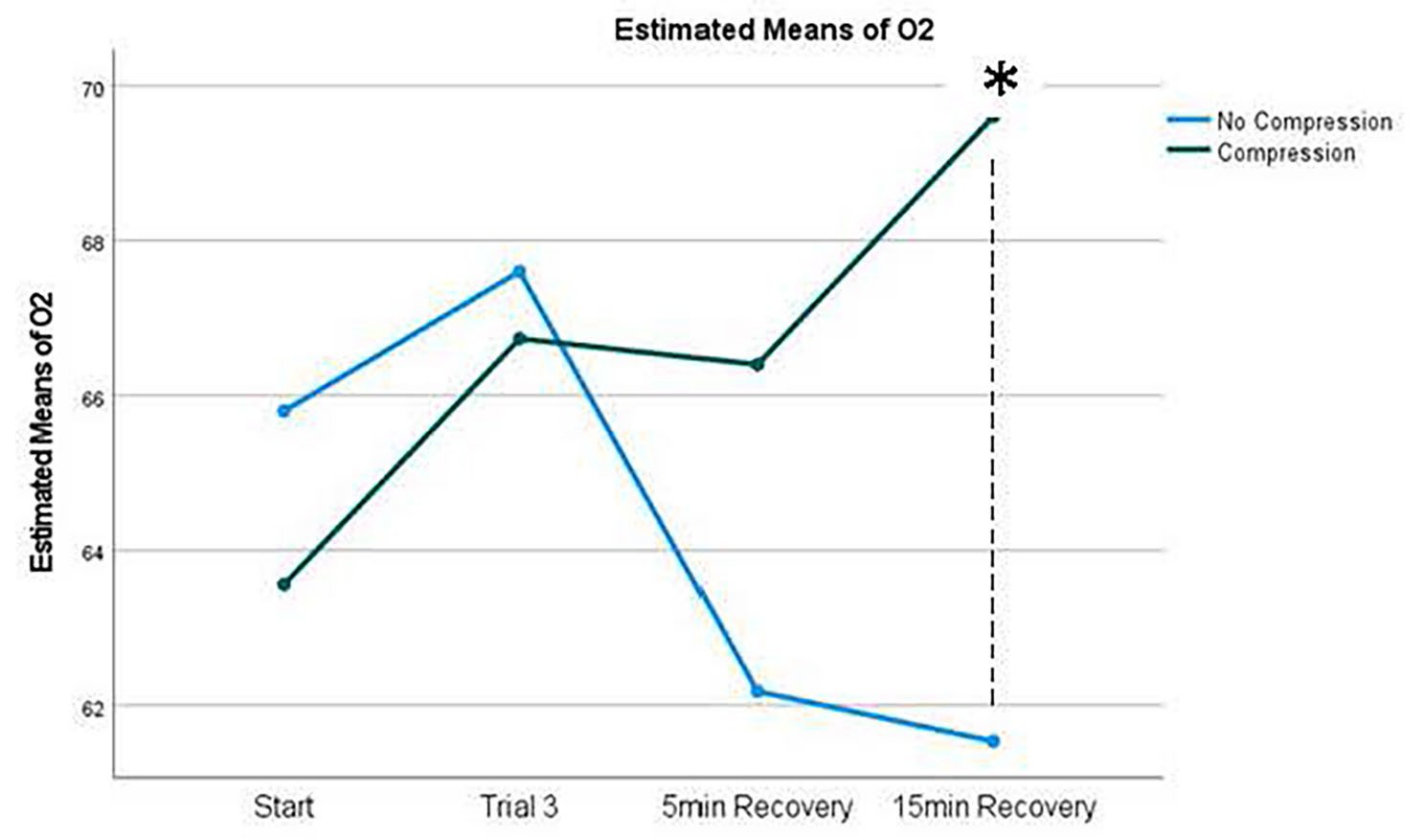
Sm02 changes in extensor carpi radialis longus *Significance The higher the Sm02, the less fatigued the muscle

An increase in Sm02 demonstrates a more rapid recovery of the working muscle. There was a 4.3% increase in Sm02 following 15 min of recovery in the compression group (66.7 ± 12.0 to 69.6 ± 14.0) as compared to a 10% decrease in Sm02 in the no compression group (67.6 ± 13.2 to 61.5 ± 15.0), this was significant at p = 0.004. At 5 min recovery there was a 0.5% decrease in the compression group (66.7 ± 12.0 to 66.4 ± 14.6) compared to an 8.7% decrease in Sm02 in the no compression group (67.6 ± 13.2 to 62.2 ± 14.7). However, this change was not significant at p = 0.12. (Fig. 3)

### Performance

**Fig. 4:**
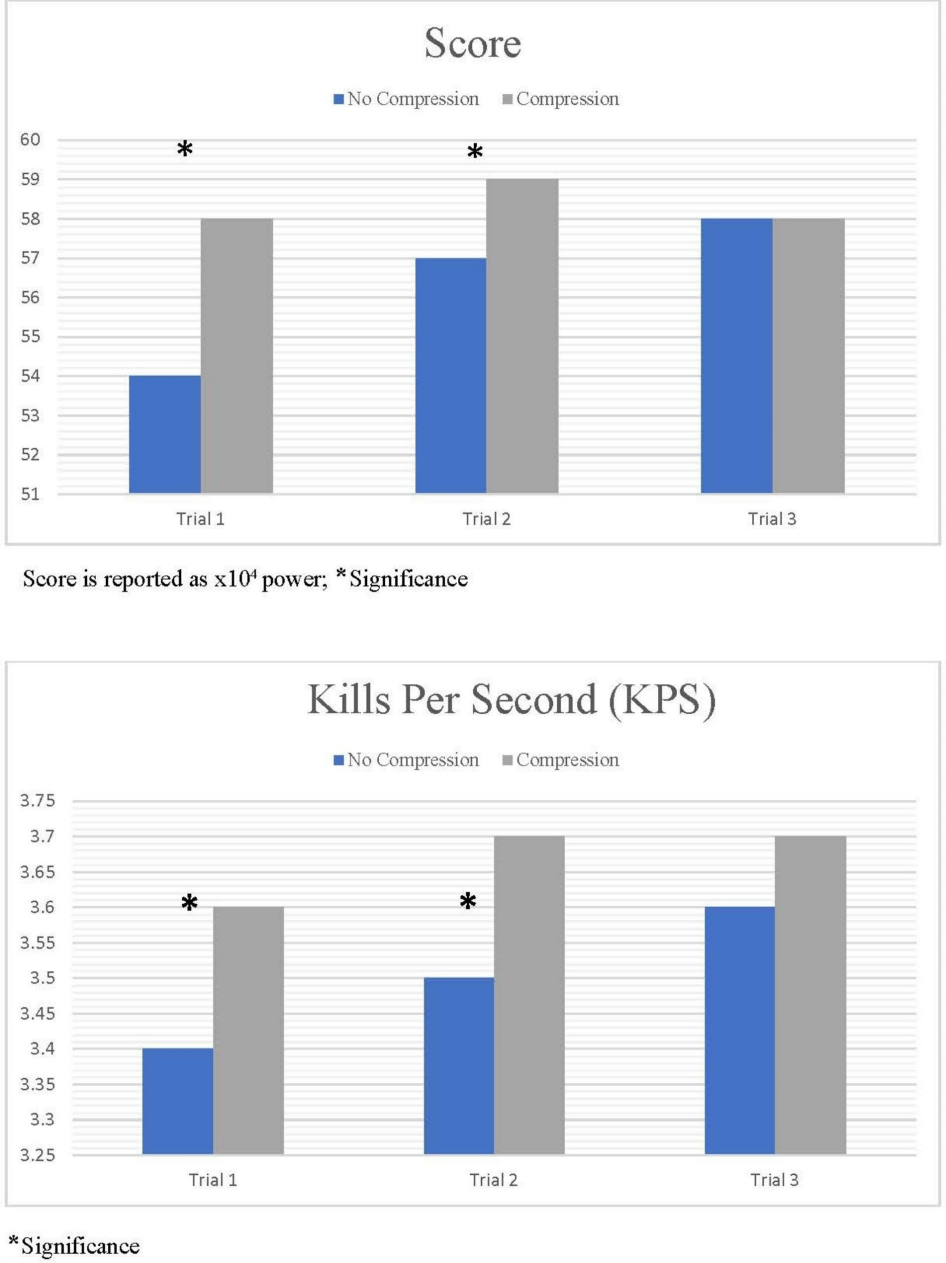
Changes in Overall Score and Kills Per Second (KPS)

There was a significant difference of overall scores and KPS in trials 1 and 2 for compression compared to no compression (p = 0.002, 0.006), with no change by the 3rd trial. Total time to kill (TTK) was faster in all of the compression trials, however the difference did not reach significance (p = 0.06). There was no change in Accuracy (p = 0.24). (Fig. 4)

### Exit survey findings

The study also measured qualitative data utilizing a mixed methods research approach. Regarding the use of the compression garments, 46.7% of participants perceived that the arm compression sleeve “positively helped” gaming performance (Table 3). 26.7% of participants perceived that it “negatively impacted” on performance, while 26.7% perceived that it had no impact on performance. Most participants who perceived that the compression sleeve positively helped gaming performance commonly noted that it provided stabilization and “less burn”. Other comments included “I felt more supported” or “my arm felt warmer”.

**Table 3:** Study exit survey outcomes

How did compression impact performance?	
Negatively	4 (26.7%)
No Impact	4 (26.7%)
Positively	7 (46.7%)
If instructed by a professional or coach to wear would you?	
No	1 (6.7%)
Yes	14 (93.3%)
I enjoyed wearing the compression	
Disagree	2 (13.3%)
Neutral	5 (33.3%)
Agree	8 (53.3%)
I would consider wearing compression in future	
Disagree	2 (13.3%)
Neutral	0 (0.0%)
Agree	13 (86.7%)
Color and look matters to me	
Disagree	7 (46.7%)
Neutral	2 (13.3%)
Agree	6 (40.0%)
I would wear compression if part of my team uniform	
Disagree	0 (0.0%)
Neutral	1 (6.7%)
Agree	14 (93.3%)

Participants who perceived that the compression sleeve negatively impacted performance noted that it “felt too tight”, “the fabric was itchy” and, of note, that the fabric did not glide smooth on the mouse pad and felt like it was sticking.

When posed the statement, “I would consider wearing compression in the future”, over 85% agreed that they would consider wearing it, while over 90% agreed that they would wear it if it were part of a team uniform.

## Discussion

The purpose of this study was to investigate if wearing a compression sleeve below the elbow affected muscle tissue oxygenation during and after high-intensity FPS aim training. One major finding was a considerable rise in Sm02 observed after 15 min of recovery while wearing the compression sleeve, as well as an improvement in performance. An interesting finding from this study was that the wearing of arm compression garments positively affected performance during an FPS training task. This is the first study to show that upper compression sleeves had a favorable effect on a high-intensity gaming activity. Several theories and mechanisms can account for these findings.

### Performance

In this current study, the players while wearing the compression sleeve showed significant improvement in performance within the first 8 min of training which continued through 16 min of training, despite negligible changes in Sm02 and HR. This is in line with previous research that demonstrated cycling improvement with compression [12, 20].

Applied pressures between 8 and 30 mm Hg to a local area have been shown to significantly increase blood flow to the underlying tissue [1, 21, 22]. Bochmann et al. [16] found an external compression to the forearm ranging from 13 to 23 mmHg significantly increased arterial perfusion more than two-fold. The rationale for this is thought to be a regulatory response that occurs following a decrease in transmural vascular pressure, which in turn triggers a myogenic response. A myogenic response is a reflex response (due to the compression) that changes blood pressure and vascular wall tension which would relax vessels, resulting in an increased blood flow [16]. Increased blood flow may delay exhaustion of the muscle by increasing intramuscular pressure which increases recruitment of motor units and delays exhaustion or fatigue while performing an activity [15, 23].

The current evidence on compression sleeves and various forms of exercise performance is conflicting. For example, Dascombe and colleagues [24] found lower body compression garments did not correspond to any improvement in running endurance in well-trained runners; similarly, Scanlan et al. [10] found no change in endurance wearing lower leg compression garments on well-trained cyclists. Whereas other data shows improvement in muscle blood flow and performance during repeated sprint cycling and time trial performances [12, 20], data on lower limb compression on performance during jumping, sprinting, or prolonged running or cycling show little to no benefit wearing compression garments [23]. Kerherve et al. [11] found that using a calf compression sleeve did not change running performance, muscle SM02 or heart rate. However, they did find that compression changed running biomechanics to be more efficient, which in turn improved their perception of pain in the Achilles tendon while trail running. Prior researchers proposed that compression may act upon skin receptors which, in turn, may enhance proprioception and may reduce muscle oscillation [25, 26]. This was noted by some participant responses stating that the compression sleeves made them feel more supported. However, this may also be explained by a placebo effect which is difficult to control for. Nevertheless, placebo effects that benefit the player can still be beneficial to performance.

### Recovery

Recovery methods in traditional sports are highly specific to the type of exercise, the intensity, and the duration. There are many extrinsic factors in lieu of or in conjunction with gaming that can cause fatigue and microtrauma with repetitive motion. These may include, but not limited to, poor posture, poor technique, the types of surfaces and accessories being used, and malalignment and muscle imbalance [27]. Overuse injuries can lead to chronic muscular inflammation or chronic degeneration of muscle and tendons. Acutely, if a muscle is fatigued, it can impact grossly on performance. These intense long hours of repetitive overuse have left the esport world struggling for ways to improve recovery, decrease acute injuries and prevent chronic overuse injury while maintaining a competitive edge. Some recovery methods used in traditional sports include ice baths, contrast baths, massage and even light exercise [27].

A strong finding from this study was the improvement in muscle reoxygenation and recovery while wearing arm compression sleeves. At 15 min recovery, arm compression displayed an improvement in muscle reoxygenation compared to not wearing compression sleeves. This reoxygenation was 9.4% higher than resting Sm02 values. This may be explained by the intensity of the activity. After vigorous and moderate exercise, it is common for oxygen saturation to rise above the resting level [28].

Due to the repetitive nature of the activity necessary for FPS shooting, individual muscle groups may become fatigued. As a result, increased perfusion and blood flow may result in increased oxygenation and a faster washout of metabolic products. Thus, edema, muscular discomfort, and muscle injury may be reduced [15]. More importantly, higher performance is often the result of increased muscular recuperation [27].

### Limitations

Prior literature concludes that the benefits of compression clothing seem to be most pronounced when it is applied for recovery purposes 12 to 48 h after significant amounts of muscle-damage-inducing exercise [27]. This study, however, only observed acute effects. The muscle studied is only part of the muscular system associated with hand, wrist and arm movements associated with video gaming, as some of the other muscles involved were unable to be measured using near infrared spectrometry due to the current technology. Therefore, this study did not fully investigate the changes to all the muscles associated with mouse gaming devices. Further research should evaluate the effect of compression on other muscles, long-term recovery, and injury.

Some studies suggest that wearing compression garments at rest and at the start of exercise increases skin blood flow which may elicit an erroneous reading of increased Sm02 [29, 30]. However, the influence of skin blood flow on 02 levels is minimal and Bochmann et al. [16] found no change in skin temperature when assessing forearm compression. However, not all compression sleeves are created equal. It should be noted that there are a variety of compression gear to choose from commercially and medically. The fabric and material used in compression garments can vary and this component is rarely noted in studies. Materials used can influence subject comfort, heat retention and moisture. During this study, the forearm compression sleeve used was made up of 73% nylon and 27% lycra and averaged a distal compression of 24(5.3) mmHg and 16(3.6) mmHg proximal compression. Future research should focus on the differences in compression levels and different types of materials. Finally, the placebo effect of wearing the compression garment was not taken into account in this investigation.

### Practical applications

This study provides support for the hypothesis that wearing upper body compression sleeves while performing high intensity video gaming may reduce fatigue, improve muscle oxygen recovery, and improve gaming performance. The improvement in video game performance suggests that, while it could be attributable to either a psychosomatic or physiological response to the compression sleeve, there is a link between wearing compression and perceived comfort while gaming. This study also provides evidence for the use of compression sleeves to re-oxygenate fatigued muscles following high intensity gaming. It is important to note that this data was collected in subjects who video gamed regularly and played competitively. In less experienced or trained video gamers, Sm02 results may differ.

Based on the current study, upper body compression wear has positive effects on acute performance and recovery following high intensity video gaming. We recommend the application of upper body compression for recovery in competitive esport players’ who tolerate it well, however further research is warranted on upper body sleeve compression and video game play that can distinguish between different levels of compression and materials under various conditions.

## Data Availability

The datasets generated and/or analyzed during the current study are not publicly available due to limited resources but are available from the corresponding author on reasonable request.

## Abbreviations

APM: Action Moves per Minute
KPS: Kills Per Second
O2: Oxygen
Sm02: Muscle Oxygen Saturation
TTK: Time To Kill
